# Support for the higher-order factor structure of the WHODAS 2.0 self-report version in a Dutch outpatient psychiatric setting

**DOI:** 10.1007/s11136-021-02880-8

**Published:** 2021-06-12

**Authors:** Guido L. Williams, Edwin de Beurs, Philip Spinhoven, Gerard Flens, Muirne C. S. Paap

**Affiliations:** 1 Dimence Foundation, Specialized Assessment and Treatment Division, Department of Digital Mental Healthcare, Dimence Group, Deventer, The Netherlands; 2 Institute of Psychology, Leiden University, Leiden, The Netherlands; 3 Department of Psychiatry, Leiden University Medical Center, Leiden, The Netherlands; 4 Alliance for Quality in Dutch Mental Health Care, Akwa GGZ, Utrecht, The Netherlands; 5 Nieuwenhuis Institute for Educational Research, Faculty of Behavioural and Social Sciences, University of Groningen, Groningen, The Netherlands; 6 Clinic Mental Health and Addiction, Oslo University Hospital, Oslo, Norway

**Keywords:** WHODAS 2.0, Functioning, Measurement, Dimensionality, Norm scores, Mental healthcare

## Abstract

**Purpose:**

Previous studies of the World Health Organization Disability Assessment Schedule 2.0 (WHODAS 2.0) interview version suggested a second-order model, with a general disability factor and six factors on a lower level. The goal of this study is to investigate if we can find support for a similar higher-order factor structure of the 36-item self-report version of the WHODAS 2.0 in a Dutch psychiatric outpatient sample. We aim to give special attention to the differences between the non-working group sample and the working group sample. Additionally, we intend to provide preliminary norms for clinical interpretation of the WHODAS 2.0 scores in psychiatric settings.

**Methods:**

Patients seeking specialized ambulatory treatment, primarily for depressive or anxiety symptoms, completed the WHODAS 2.0 as part of the initial interview. The total sample consisted of 770 patients with a mean age of 37.5 years (*SD* = 13.3) of whom 280 were males and 490 were females. Several factorial compositions (i.e., one unidimensional model and two second-order models) were modeled using confirmatory factor analysis (CFA). Descriptive statistics, model-fit statistics, reliability of the (sub)scales, and preliminary norms for interpreting test scores are reported.

**Results:**

For the non-working group, the second-order model with a general disability factor and six factors on a lower level, provided an adequate fit. Whereas, for the working group, the second-order model with a general disability factor and seven factors on a lower level seemed more appropriate. The WHODAS 2.0 36-item self-report form showed adequate levels of reliability. Percentile ranks and normalized T-scores are provided to aid clinical evaluations.

**Conclusion:**

Our results lend support for a factorial structure of the WHODAS 2.0 36-item self-report version that is comparable to the interview version. While we conjecture that a seven-factor solution might give a better reflection of item content and item variance, further research is needed to assess the clinical relevance of such a model. At this point, we recommend using the second-order structure with six factors that matches past findings of the interview form.

**Supplementary Information:**

The online version contains supplementary material available at 10.1007/s11136-021-02880-8.

Adequate levels of functioning play a key role in successful aging in regard to deriving meaning and purpose in life, and have various favorable effects on mental and physical health [1, 2] According to the definition of the World Health Organization (WHO), health is “a state of complete physical, mental and social well-being and not merely the absence of disease or infirmity.” In line with this definition, it is safe to assume that merely using a medical classification and an inventory of symptoms, yield insufficient information on the level of care that is needed and the expected outcomes of healthcare. Patients do not only seek treatment for symptom relief, but often also want to resolve interpersonal problems and learn to cope with their struggles in daily functioning and (social) participation, caused by their (mental) illness. Furthermore, collecting data on the level of functioning could play an important role in the planning, monitoring, evaluation, and outcome of healthcare [3].

The International Classification of Functioning, Disability and Health (ICF), published by the WHO, offers a standardized method for integrating the level of functioning in a diagnosis [4]. The ICF offers (coded) descriptions of the level of functioning in body functions (including mental functions), body structures, daily activities and (social) participation. The World Health Organization Disability Assessment Schedule 2.0 (WHODAS 2.0) [5], which is directly connected to the ICF concepts, is recommended by the WHO and the International Consortium for Health Outcomes Measurement (ICHOM) as a standard measure for the level of functioning [6]. The Diagnostic and Statistical Manual of Mental Disorders, Fifth Edition (DSM-5) by the American Psychiatric Association (APA), also promotes the WHODAS 2.0 as the successor of the Global Assessment of Functioning (GAF), in order to provide a more accurate measure of the global level of disability in psychiatric patients [7, 8].

The WHODAS 2.0 was based on a large cross-cultural study, encompassing samples from the general population, populations with physical problems, populations with mental or emotional problems, and populations with problems related to alcohol and drug use [8–10]. The WHODAS 2.0 was found to have good psychometric properties across all included samples. Construct validity of the scales was evaluated by conducting principal components analysis and the item responses were analyzed for ordinality applying the partial credit model. Finally, 34 items of the originally 96 proposed items were eligible for inclusion in the WHODAS 2.0, and two additional items concerning sexual activity and the impact of the disability on the family, were incorporated in the 36-item version. From exploratory factor analysis, the researchers suggested a second-order model, with a general disability factor and six factors on a lower level in different cultures and populations which was replicated by conducting confirmatory factor analysis [9].

For the initial development of the WHODAS 2.0, an interview version was used. However, in clinical practice it might be convenient and efficient to make use of a self-report version. Some authors have suggested that self-evaluations could give a more accurate measure of patient symptoms in psychiatric assessments as they reduce the risk of bias on the part of the clinicians [10]. Furthermore, there are indications that for some symptoms, ratings by patients seem to be more conservative than ratings by proxy; i.e., patients tend to report fewer symptoms than clinicians or family [3, 11]. Still, studies on the self-report version seem to be scarce. To our knowledge there are no self-report studies of the full 36-item self-report Dutch WHODAS examining the internal structure and/or providing norms for interpreting the scores for the general Dutch population or Dutch psychiatric population. An international systematic review on the use and psychometric properties of the WHODAS 2.0 [10] identified 810 studies from 94 countries published between 1999 and 2015, using the WHODAS (i.e., the 36-item version, 12-item version or the combined 12 + 24 item version in various formats: interview-administered, self-administered, and/or proxy-administered). From this review it is not clear how many studies used the 36-item patient self-report (i.e., self-administered) version specifically; but only fifteen of these studies were conducted in the field of psychiatry in The Netherlands using a 36-item format. However, these Dutch studies did not examine the psychometric properties of the WHODAS, but mostly used the WHODAS as an independent measure of functional impairment. Only two of these studies reported on reliability (Cronbach’s Alpha), and one study reported a mean score with a standard deviation for the WHODAS. For these reasons, it should be evaluated whether the factor structure of the WHODAS 2.0 interview version is also adequate for the self-report version.

In addition, some sample characteristics of the initial international field studies [5] raise questions regarding the generalizability of these field studies to the population of psychiatric patients [12]. According to the WHODAS 2.0 manual, 27.6% and 25.7% of the total international sample (*N* = 1431), respectively, had mental or emotional problems [5]. The proportion of Dutch participants in the total international sample was relatively small (WHODAS 2.0 field studies: Item reduction and feasibility *n* = 47, i.e., approx. 3% of the total sample; WHODAS 2.0 field studies: Reliability and validity *n* = 50, i.e., approx. 3.5% of the total sample). It is therefore important to address the generalizability of the WHODAS 2.0; in particular, regarding the group of Dutch psychiatric patients that participated in our research. Moreover, the Dutch translation of the DSM-5 was already published in 2014, including the Dutch WHODAS 2.0 36-item self-report as a replacement for the GAF [13]. Since 2017, a DSM-5 classification has been mandatory in the Dutch mental health care system in order to be eligible for reimbursement for treatments by health insurance companies. However, due to the lack of Dutch psychometric data, clinicians have chosen to continue using the outdated GAF. Meanwhile, the Dutch Healthcare Institute, responsible for formulating national healthcare quality standards and treatment guidelines, also favors the WHODAS 2.0 as a measure for the level of functioning in psychiatric patients [14].

The main aim of this study is to investigate whether the original factor structure (i.e., a second-order model, with a general disability factor and six factors on a lower level), is supported for the Dutch self-report version. We also focus on the differences between the non-working group sample and the working group sample, because non-working is hypothesized to be associated with higher scores on the WHODAS 2.0 i.e., higher levels of disability. In addition, we provide preliminary norm data to allow for interpretation of WHODAS 2.0 scores in psychiatric settings.

## Method

### Data collection

The patients in this study were referred by their general practitioner to the Ambulatory Specialized Assessment and Treatment Division of the Dimence foundation from the Dimence Group (DG), a Dutch mental health care facility for psychiatric disorders. After referral, the patients were invited for an initial diagnostic interview by at least one (specialized) healthcare professional. After the initial interview, patients received a primary diagnosis. Before or after this appointment, the patients filled out the WHODAS 2.0 36-item self-report form on a computer, as part of the standard assessment during the admission process. For this study, all patients who completed the WHODAS 2.0 within a 50-day window (i.e., before/after the initial interview) were included. The raw, coded, WHODAS 2.0 scores were obtained from the data warehouse of the DG by a senior business intelligence developer of the Business Intelligence Team. Besides the item scores, the following demographic data were also available from the database: gender, diagnosis, age, treatment region, education level and date of completion of the WHODAS 2.0.

### Materials

The WHODAS 2.0 self-report version contains 36 items for assessing problems related to (mental) health conditions experienced during the past 30 days. Ratings were given on a 5-point Likert-scale with higher scores reflecting higher levels of disability: (1) none, (2) mild, (3) moderate, (4) severe, (5) extreme.

The WHODAS 2.0 produces a mean score^1^ for all items and for each of the six subscales of functioning domains: D1 cognition (6 items; e.g., “Concentrating on doing something for ten minutes”); D2 mobility (5 items; e.g., “Standing up from sitting down”); D3 self-care (4 items; e.g., “Getting dressed”); D4 getting along (5 items; e.g., “Maintaining a friendship”); D5 life activities (household: 4 items; e.g., “Taking care of your household responsibilities;” and work/school: 4 items; e.g., “Your day-to-day work/school”); and D6 participation (8 items; e.g., “How much of a problem did you have because of barriers or hindrances in the world around you?”).

It should be noted that the life activities domain D5 of the WHODAS 2.0 contains two sub-clusters: household (4 items) and work/school activities (4 items). Respondents who do not participate in work- or school-related activities are instructed to skip the corresponding items. This could lead to relatively lower overall disability scores for the non-working group, compared to respondents participating in work- or school-related activities who are reporting difficulties in this domain [15]. Therefore, the scores of the working group and the non-working group could not be directly compared, and for that reason we calculated mean scores instead of sum scores.

Over the past few years, a number of international initiatives have advocated the conversion of raw scores into T-scores in an effort to harmonize measurements results and to facilitate the comparability and ease of interpretation across different measurement instruments [6, 16–18]. For that reason, we included a conversion table to convert raw mean scores into T-scores (table S8).

### Analysis

All analyses were performed in R, version 3.6.1 [19]. The following additional packages were used: lavaan version 0.6.5. [20]; tidyverse version 1.2.1. [21]; psych version 1.8.12. [22]; semTools version 0.5.2.916 [23] and semPlot version 1.1.2. [24]; CTT version 2.3.3. [25]; and effsize version 0.7.8. [26].

We described the characteristics of the psychiatric population. In addition, we assessed the dimensionality conducting confirmatory factor analysis (CFA). Due to the missing values for the non-working group in the life activities domain D5, the CFAs were performed separately for the working group (36 items) and non-working group (32 items, with scale D5 being defined as D5.1 + D5.2 + D.5.3 + D5.4). Additionally, to analyze the effect of the missing values in the non-working group, we conducted a CFA for all models on the total sample with imputation. For this calculation we used the mean scores of the working sample as proposed by the WHODAS 2.0 manual [5].

We compared three models to investigate the factorial structure of the WHODAS 2.0:

1. A unidimensional model. Here, all items were treated as indicators where all items loaded on one single factor.
2. A second-order factor model using 6 subscales. The association among the subscales was modeled by adding a second-order factor. This factor structure is suggested as the preferred model for the initial interview version of the WHODAS 2.0 by Üstün et al. [9].
3. A second-order factor model using 7 subscales for the working group. For this model we added a factor by splitting subscale 5 (life activities) into two different components (household and work/school activities). This model could only be applied to the working group and the imputed sample.

For the sake of completeness, additional information regarding correlated trait models with six and seven factors respectively is available in Online Supplement A.

We measured reliability by calculating Omega. We chose to favor Omega over Cronbach’s Alpha since Omega gives a more accurate estimate of reliability in case of a higher order structure [27–30]. Although Omega and Cronbach’s Alpha can yield different (or similar) results, the general criteria for interpretation apply to both. Reliability estimates of 0.80 and higher were considered sufficient, following recommendations of Nunnally and Bernstein [31], and Lance et al. [32].

Since the items were ordinal in nature, we used the weighted least square mean and variance (WLSMV) estimator [33, 34]. The WLMSV estimator in lavaan, a package in R, which is used for structural equation modelling including CFA, uses diagonally weighted least squares (DWLS) with robust standard errors and a mean- and variance-adjusted test statistic, to estimate the model parameters with the full weight matrix based on polychoric correlations, using the ‘ordered’ argument [20]. The factor loading of the first indicator of each latent variable was fixed to 1. The following CFA fit statistics were considered to be indicative of good fit [35, 36]: comparative fit index (CFI) > 0.95, Tucker–Lewis index (TLI) > 0.95, root mean square error of approximation (RMSEA) < 0.06, standardized root mean square residual (SRMR) < 0.08.

## Results

### Descriptive statistics

The total sample consisted of 770 patients from the Specialized Assessment and Treatment Division of the Dimence foundation from the Dimence Group. The total sample had a mean age of 37.5 years (*SD* = 13.3, range 17—67) and consisted of 280 males and 490 females (with a mean age of 39.59 (*SD* = 13.06) and 36.31 (*SD* = 12.99) years, respectively.

The majority of participants (70%) had a primary diagnosis of a depressive disorder or an anxiety disorder. For 123 participants (16%), the primary diagnosis was unknown, because the psychological evaluation was not fully completed at the time of our data collection. Most of the participants, *n* = 483 (i.e., 63%), were employed (paid, non-paid, self-employed) or went to school. All participants resided in the central-eastern regions of The Netherlands. The mean score for the total sample (*N* = 770) was 2.42 (*SD* = 0.71, skewness = 0.45, kurtosis = − 0.19, se = 0.03). More detailed demographics and descriptive statistics are presented in Tables 1 and 2.

**Table 1:** Demographic characteristics total sample (*N* = 770)

Variable	*M* (*SD*) range, *n* [%]
Age (years)	37.5 (13.1) range 17–67
Male	39.6 (13.1) range 18–67
Female	36.3 (13.0) range 17–63
Sex	
Male	280 [36.4%]
Female	490 [63.6%]
Education level	
Low	39 (Male = 16 [2%], Female = 23 [3%])
Middle	474 (Male = 166 [22%], Female = 308 [40%])
High	218 (Male = 76 [10%], Female = 142 [18%])
Unknown	39 (Male = 22 [3%], Female = 17 [2%])
Working/school	
Yes	*N* = 483 [63%] (Male = 183 [24%], Female = 300, [39%])
No	*N* = 287 [37%] (Male = 97 [12%], Female = 190 [25%])
Primary diagnosis	
Depressive disorders	308 (Male = 124, Female = 184)
Anxiety disorders	225 (Male = 67, Female = 158)
Unknown	123 (Male = 49, Female = 74)
Personality disorders	70 (Male = 18, Female = 52)
Neurodevelopmental disorders	14 (Male = 9, Female = 5)
Somatic symptom and related disorders	13 (Male = 5, Female = 8)
Substance-related and addictive disorders	8 (Male = 5, Female = 3)
Schizophrenia spectrum and other psychotic disorders	4 (Male = 1, Female = 3)
Feeding and eating disorders	2 (Male = 0, Female = 2)
Disruptive, impulse control, and conduct disorders	2 (Male = 2, Female = 0)
Dissociative disorders	1 (Male = 0, Female = 1)

**Table 2:** Descriptive statistics mean scores WHODAS 2.0 for working, non-working and total sample

Domain	Working (*n* = 483)					Non-Working (*n* = 287)					Total Sample (*n* = 770)				
	*M*	SD	Skew	Kurtosis	se^*a*^	*M*	SD	Skew	Kurtosis	se^*a*^	*M*	SD	Skew	Kurtosis	se^*a*^
D1	2.52	0.92	0.40	− 0.52	0.04	2.77	0.96	0.19	− 0.82	0.06	2.61	0.94	0.32	− 0.66	− 0.03
D2	1.83	0.91	1.15	0.41	0.04	3.33	1.15	0.19	− 0.82	0.07	2.02	1.01	0.90	− 0.24	0.04
D3	1.74	0.79	1.27	1.27	0.04	2.03	0.96	0.96	0.26	0.06	1.85	0.86	1.18	0.93	0.03
D4	2.41	1.00	0.41	− 0.74	0.05	2.65	1.04	0.26	− 0.91	0.06	2.50	1.02	0.36	− 0.80	0.04
D5	2.48	1.13	0.43	− 0.82	0.05	2.88	1.26	0.11	− 1.13	0.07	2.76	1.01	0.19	− 0.87	0.05
D6	2.69	0.86	0.20	− 0.52	0.04	3.01	0.90	− 0.11	− 0.68	0.05	2.81	0.89	0.09	− 0.64	1.17
Total	2.08	0.62	0.54	− 0.08	0.03	2.67	0.77	0.24	− 0.44	0.05	2.42	0.71	0.45	− 0.19	0.03

^a^Standard error of mean

An independent samples *t*-test was conducted to compare the mean scores for the working group and non-working group. There was a significant difference in the mean scores between these groups; *t* (506.83) = 10.979, *p* < 0.001. The effect size for this analysis (*d* = 0.86; 95% CI [0.71–1.02]) was found to exceed the convention for a large effect (*d* = 0.80) [37]. This indicates that the mean score for the average patient in the non-working group is 0.86 standard deviation above the mean score for the average patient in the working group.

On examining the distribution of the response categories endorsed per item (see Online Supplement B), it was found that over 55% of the respondents chose scoring category 1 (indicating no disability) for the mobility items D2.2, D2.3 and D2.5. More than 70% of the respondents also reported no difficulties with the self-care items D3.1 and D3.2. Interestingly, the participation item D6.6 also was highly positively skewed, as more than half of the respondents indicated no drain on their financial resources due to their health condition, and almost 20% experienced mild financial difficulties. However, still about a third of all patients reported moderate to extreme problems in relation to their financial situation.

### Latent variable modelling

The unidimensional model did not meet the recommended criteria for a good fit. The fit indices for the multidimensional models were acceptable. We interpreted this as support for the contention that a multidimensional model should be preferred, and that the subscales of the WHODAS 2.0 patient self-report have added value when measuring the level of functioning.

For the non-working group, the second-order model with a general disability factor and six factors on a lower level, as found in the interview version of the initial WHODAS 2.0 studies, provided an acceptable fit (Table 3). The correlated six factor model showed comparable fit (see Online Supplement A).

**Table 3:** Confirmatory factor analysis (CFA) fit indices non-working sample (*n* = 287)

Model	${X}^{2}$	p-value	*df*	$\frac{{X}^{2}}{df}$	CFI	TLI	RMSEA	SRMR
1 Factor	3705.270	< 0.001	464	7.986	0.931	0.926	0.156	0.127
2nd order 6F	1094.409	< 0.001	458	2.389	0.986	0.985	0.070	0.077

However, in the working group, not all fit statistics met the desired criteria for the second-order model with six factors. For the working group, the second-order model with a general disability factor and seven factors on a lower level seems more appropriate (Table 4). Of note, the correlated traits model with seven factors had an even marginally better fit (see Online Supplement A). This model lends support to the aforementioned notion that subscale D5 contains two sub clusters with different content. However, a comparison of the item loadings in D5 between the working and non-working groups, showed no important differences. Therefore, it seems safe to assume that the D5 items measure the same underlying concept in both the working and non-working groups (standardized coefficients for all CFA models are provided in online supplement A).

**Table 4:** Confirmatory factor analysis (CFA) fit indices working sample (*n* = 482)

Model	${X}^{2}$	p-value	*df*	$\frac{{X}^{2}}{df}$	CFI	TLI	RMSEA	SRMR
1 Factor	9111.867	< 0.001	594	15.339	0.917	0.912	0.173	0.144
2nd order 6F	3708.296	< 0.001	588	6.307	0.970	0.968	0.105	0.100
2nd order 7F	1780.579	< 0.001	587	3.033	0.988	0.988	0.065	0.069

Imputation is a suitable method for dealing with missing data that is convenient to use and easy to interpret [37]. To determine the effect of imputation of the missing values in the non-working group, we also evaluated all models by assigning the mean of 3 derived from the working group to the missing items D5.5–D5.8 in the non-working group, as proposed by the WHODAS 2.0 manual [5]. When imputing the missing D5 items in the non-working group by the mean of 3, the second-order model with seven factors performed well (Table 5). The second-order model with six factors showed mixed results. It did not meet the pre-set threshold values for the RMSEA (0.114) and SRMR (0.111); but the CFI (0.965) and TLI (0.962) values corresponded to a good fit, though marginally worse compared to the second-order model with seven factors. Here it might also be of interest to point out that a correlated traits model with seven factors could compete with the second-order model with seven factors (see online supplement A). The reliability levels of the factors, as measured by coefficient ω (Table 6), were adequate (ω > 0.80), except for the self-care factor within the working group (ω = 0.76).

**Table 5:** Confirmatory factor analysis (CFA) fit indices imputed sample (*n* = 770)

Model	${X}^{2}$	p-value	*df*	$\frac{{X}^{2}}{df}$	CFI	TLI	RMSEA	SRMR
1 Factor	16,658.644	< 0.001	594	28.04	0.904	0.898	0.188	0.161
2nd order 6F	6472.337	< 0.001	588	11.01	0.965	0.962	0.114	0.111
2nd order 7F	2574.417	< 0.001	587	4.386	0.988	0.987	0.066	0.065

**Table 6:** Omega reliability values CFA

	Omega ω		
	Working	Non-Working	Imputed^b^
1 Factor	0.95	0.95	0.95
6 Factor			
Cognition	0.85	0.85	0.85
Mobility	0.86	0.87	0.87
Self-care	0.76	0.84	0.80
Getting along	0.82	0.81	0.82
Life activities	0.96	0.94	0.94
Participation	0.84	0.85	0.85
7 Factor		N/A	
Cognition	0.85		0.85
Mobility	0.86		0.87
Self-care	0.76		0.80
Getting along	0.82		0.82
Household	0.93		0.94
Work	0.95		0.93
Participation	0.84		0.85
2nd order 6F	0.85 (ωL1^a^)	0.86 (ωL1^1^)	0.85 (ωL1^a^)
Cognition	0.85	0.85	0.85
Mobility	0.86	0.87	0.87
Self-care	0.76	0.84	0.80
Getting along	0.82	0.81	0.82
Life activities	0.96	0.94	0.95
Participation	0.84	0.85	0.85
2nd order 7F	0.88 (ωL1^a^)	N/A	0.87(ωL1^a^)
Cognition	0.85		0.85
Mobility	0.86		0.87
Self-care	0.76		0.80
Getting along	0.82		0.82
Household	0.93		0.94
Work	0.95		0.93
Participation	0.84		0.85

^a^Proportion of the second-order factor explaining the score, or the coefficient omega at Level 1

^b^Imputation by a mean of 3 in the non-working group for items D5.5–D5.8

### Norms

Percentile ranks and T-scores (*M* = 50, *SD* = 10) for the mean scores were calculated (i.e., for all items and the 6 subscales) as shown in Tables 7 and 8. To illustrate, for the working group a mean score between 1.44 and 2.72 equals normalized T-scores between 40 and 60, which are considered moderate or average. Working patients with T-scores above this range (i.e., 1 *SD* > mean) might signal severe disability; patients with a T-score above 70 (i.e., 2 *SD* > mean) are likely to show extreme disability.

**Table 7:** Mean scores and corresponding percentile ranks WHODAS 2.0

Percentile	0%	5%	20%	40%	60%	80%	95%
Label Domain	None	Mild	Mild/Moderate	Moderate	Moderate/Severe	Severe	Extreme
Mean total score							
Working	0.89	1.14	1.53	1.86	2.14	2.58	3.19
Nonworking	1.00	1.42	2.00	2.41	2.86	3.31	4.00
Total (*n* = 482)	1.00	1.39	1.75	2.21	2.53	3.00	3.69
D1_Cognition							
Working	1.00	1.17	1.67	2.17	2.67	3.33	4.17
Nonworking	1.00	1.33	1.83	2.50	3.00	3.67	4.45
Total (*n* = 770)	1.00	1.67	1.67	2.33	2.83	3.50	4.67
D2_Mobility							
Working	1.00	1.00	1.00	1.20	1.80	2.60	3.80
Nonworking	1.20	1.60	2.20	3.00	3.60	4.40	5.34
Total (*n* = 770)	1.00	1.00	1.00	1.40	2.00	3.00	4.11
D3_Self-care							
Working	1.00	1.00	1.00	1.25	1.75	2.25	3.25
Nonworking	1.00	1.00	1.25	1.50	2.00	3.00	4.00
Total (*n* = 770)	1.00	1.00	1.00	1.50	1.75	2.50	3.75
D4_Getting along							
Working	1.00	1.00	1.40	2.00	2.60	3.40	4.64
Nonworking	1.00	1.00	1.80	2.20	2.80	3.76	4.40
Total (*n* = 770)	1.00	1.00	1.56	2.00	2.68	3.40	4.20
D5_Life activities							
Working	1.00	1.00	1.25	2.00	2.75	3.50	4.50
Nonworking	1.00	1.00	1.75	2.50	3.00	4.00	5.00
Total (*n* = 482)	1.00	1.00	1.56	2.00	2.68	3.40	4.80
D6_Participation							
Working	1.00	1.38	1.88	2.50	2.88	3.50	4.13
Nonworking	1.00	1.54	2.13	2.75	3.38	3.88	4.46
Total (*n* = 770)	1.00	1.38	2.00	2.50	3.00	3.63	4.25

**Table 8:** Conversion table of raw mean scores to corresponding T-scores WHODAS 2.0

	T-scores				
	30 None	40 Mild	50 Moderate	60 Severe	70 Extreme
Mean total score					
Working	1.03	1.44	2.00	2.72	3.56
Nonworking	1.31	1.88	2.63	3.50	4.28
Total (*n* = 482)	1.22	1.67	2.36	3.17	4.06
D1_Cognition					
Working	1.17	1.67	2.50	3.50	4.50
Nonworking	1.17	1.83	2.67	3.83	4.67
Total (*n* = 770)	1.17	1.67	2.50	3.67	4.67
D2_Mobility					
Working	N/A	1.00	1.60	2.80	4.20
Nonworking	1.40	2.00	3.20	4.60	5.60
Total (*n* = 770)	N/A	1.00	1.80	3.20	4.40
D3_Self-care					
Working	N/A	1.00	1.50	2.50	3.75
Nonworking	N/A	1.25	1.75	3.00	4.25
Total (*n* = 770)	N/A	1.00	1.50	2.75	4.00
D4_Getting along					
Working	N/A	1.40	2.40	3.60	4.40
Nonworking	N/A	1.60	2.60	3.80	4.60
Total (*n* = 770)	N/A	1.40	2.40	3.60	4.60
D5_Life activities					
Working	N/A	1.25	2.25	4.00	4.75
Nonworking	N/A	1.50	2.75	4.25	N/A
Total (*n* = 482)	1.00	1.63	2.75	3.88	4.75
D6_Participation					
Working	1.13	1.75	2.63	3.63	4.50
Nonworking	1.13	2.00	3.00	4.00	4.63
Total (*n* = 770)	1.13	1.88	2.75	3.75	4.63

## Discussion

This study describes the factor structure of the WHODAS 2.0 self-report version in a Dutch psychiatric outpatient sample. Particular attention has been given to the differences between the samples of the non-working group and the working group, as non-working was hypothesized to be associated with higher scores on the WHODAS 2.0 i.e., higher levels of disability. Characteristics of the psychiatric population were described, and percentile ranks with T-scores were calculated to aid clinical decision making. The percentiles and T-scores reported in our study can be used to describe the relative impairment in relation to other patients with common mental disorders in ambulatory care.

We used CFA for evaluating dimensionality. The unidimensional model did not meet the recommended criteria for a good fit. Our results indicate that the multidimensional factor solution previously found for the interview version [9], can also be extended to the WHODAS 2.0 self-report version. The reliability levels of the tested factor models, as measured by coefficient ω, all exceeded 0.80, which we consider to be sufficient.

For the non-working group, the second-order model with a general disability factor and six factors on a lower level, provided an adequate fit. Hence, for this group, the original factor composition of the WHODAS 2.0 interview version seems also adequate for the WHODAS 2.0 self-report version as well. Although a correlated six-factor model performed even slightly better in terms of fit (see Online Supplement A), we suggest using this second-order model based on previous findings on the factor structure of the interview version and these small differences.

However, for the working group, a second-order model with a general disability factor and seven factors on a lower level seems more appropriate. A correlated traits model with seven factors, has an even (slightly) better fit (see Online Supplement A); but there seems no compelling reason to deviate from the second-order structure that is in line with previous findings.

Conceptually, this can be explained, as the item content of subscale D5 (life activities) contains two types of items: household items (D5.1–D5.4) and work/school activities items (D5.5–5.8). Separate subscales for these subdomains could represent the item content more clearly for clinicians and patients in daily practice. For the further development of the WHODAS 2.0, it might therefore be advisable to divide subscale D5 (life activities) into two separate subscales, separating the household items from the work/school activities items [12], and to consider imputing the missing D5 items in the non-working group using the mean value of 3. However, it should be noticed that the differences in terms of fit across the various factor models are modest.

It is also worth considering if not working in itself is a sign of increased disability [12]. This seems plausible, given the differences in the mean scores between the non-working group and working group. This implies that the non-working group is relatively more impaired, or has a relatively higher level of functional disability, compared to the working group.

While the WHODAS 2.0 is considered a generic assessment instrument that can be used in both clinical and general population settings, the response patterns found in subscales D2 (Mobility) and D3 (Self-care) could be an indication that these domains are of less interest, when measuring the level of disability of psychiatric patients. This would not be an unexpected finding, since it has been found that psychiatric patients with common mental disorders are not likely to reach out to mental healthcare services, primarily because of mobility or self-care issues [38]. Nevertheless, our data show that a minority of the patients endorses the higher response categories for the aforementioned items. Therefore, these items seem valuable for assessing the more severe levels of disability. This is important, because for a generic instrument, items covering the full range of the underlying trait levels are necessary.

## Limitations

Although, our total sample size was substantial, the sample of the non-working group was relatively small, which could affect the outcome of the CFA. In addition, the majority of participants (70%) had a primary diagnosis of a depressive disorder or an anxiety disorder, and for 16% the primary diagnosis was unknown. Therefore, generalizability to other psychiatric groups—especially, those with severe functional impairment—might be limited. That being said, we deem our cross-sectional study is a valuable starting point for implementing the self-report method for measuring the level of disability in psychiatric patients.

Another limitation of this study is that, despite the fact that we have found some evidence that a seven-factor model could be an improvement, external validation is necessary to substantiate this assumption. For instance, is a seven-factor model better in differentiating patients with a positive versus negative treatment outcome? We further suggest that more research should be done on the responsiveness of the WHODAS 2.0. Finally, it is important to analyze data from the Dutch general population for establishing an agreed-upon cut-point for identifying healthy subjects.

## Conclusion

Our results lend support for a factorial structure of the WHODAS 2.0 36-item self-report version that is comparable to the interview version. Based on our results, we conclude that a multidimensional model is preferred, and that the subscales of the WHODAS 2.0 36-item self-report form have added value when measuring the level of functioning. While we conjecture that a seven-factor solution might give a better reflection of item content and item variance, further research is needed to assess the clinical relevance of such a model. At this point, we recommend using the second-order structure with six factors that matches past findings of the interview form.

In sum, we consider the WHODAS 2.0 36-item self-report form a promising measure for assessing the level of functioning in Dutch psychiatric patients, in compliance with the international recommendations of the WHO, ICHOM, DSM-5 and the guidelines of the Dutch Healthcare Institute [5–7, 14].

## Supplementary Information

Below is the link to the electronic supplementary material.

Supplementary file1 (PDF 185 KB)

Supplementary file2 (PDF 92 KB)

## Data Availability

The data that support the findings of this study are available from the Dimence Group. Note that restrictions apply to the availability of these data, which were used under license for the current study, and for that reason are not publicly available. However, data are available from the authors upon reasonable request and with permission of the Dimence Group.

## Footnotes

1 The WHO manual proposes two scoring procedures, one based on classical test theory (CTT) and one based on item response theory (IRT). Since many clinicians may not have the resources, time or knowledge to use the complex IRT scoring method, we opted for the recommended ‘simple’ CTT based scoring as a method of choice in busy clinical settings. Rather than using a sum score, however, we chose to use a mean score. The main reason for this is that sum scores are less suitable for comparisons between the working and non-working samples in our study, because these groups responded to a different number of items. Importantly, the percentage scores mentioned in the manual of the WHO seem only to apply to the more complex IRT-based scoring.

## Code availability

The R codes used for analysis are available from the corresponding author upon reasonable request.
