# Supplementary material for: Support for the higher-order factor structure of the WHODAS 2.0 self-report version in a Dutch outpatient psychiatric setting
Source: Qual Life Res. 2021 Jun 12;30(10):2939–49. doi: 10.1007/s11136-021-02880-8 (PMC8481147; doi:10.1007/s11136-021-02880-8)
Supplement: Supplementary file 1 — Supplementary file1 (PDF 185 KB) [file 11136_2021_2880_MOESM1_ESM.pdf]

Online supplement A
Fit indices and Standardized Coefficients CFA's

Title: Confirmation of the WHODAS 2.0 factor structure for the self-report version in a Dutch outpatient psychiatric setting
Journal: Quality of Life Research
Authors: Guido L. Williams, Edwin de Beurs, Philip Spinhoven, Gerard Flens, Muirne C. S. Paap
Corresponding author: Dimence Group, Dimence foundation, Specialized Assessment and Treatment Division, Department of Digital Mental Healthcare, Deventer, The Netherlands; g.williams@thubble.nl; ORCID 0000-0001-8008-5750

Table 3 Supplement
Confirmatory Factor Analysis (CFA) Fit Indices Working Sample (n = 482)

Model	X^2	*p-value*	*df*	$\frac{X^2}{df}$	CFI	TLI	RMSEA	SRMR
1 Factor	9111.867	0.001	594	15.339	0.917	0.912	0.173	0.144
**6 Factor**	**3435.659**	**0.001**	**579**	**5.934**	**0.972**	**0.970**	**0.101**	**0.096**
**7 Factor**	**1360.598**	**0.001**	**573**	**2.375**	**0.992**	**0.992**	**0.053**	**0.059**
2nd order 6F	3708.296	0.001	588	6.307	0.970	0.968	0.105	0.100
2nd order 7F	1780.579	0.001	587	3.033	0.988	0.988	0.065	0.069

Table 4 Supplement
Confirmatory Factor Analysis (CFA) Fit Indices Non-Working Sample (n = 287)

Model	X^2	*p-value*	*df*	$\frac{X^2}{df}$	CFI	TLI	RMSEA	SRMR
1 Factor	3705.270	0.001	464	7.986	0.931	0.926	0.156	0.127
**6 Factor**	**901.305**	**0.001**	**449**	**2.007**	**0.990**	**0.989**	**0.059**	**0.069**
2nd order 6F	1094.409	0.001	458	2.389	0.986	0.985	0.070	0.077

Table 5 Supplement
Confirmatory Factor Analysis (CFA) Fit Indices Imputed Sample (n = 770)

Model	X^2	*p-value*	*df*	$\frac{X^2}{df}$	CFI	TLI	RMSEA	SRMR
1 Factor	16658.644	0.001	594	28.04	0.904	0.898	0.188	0.161
**6 Factor**	**6025.203**	**0.001**	**579**	**10.41**	**0.967**	**0.965**	**0.111**	**0.107**
**7 Factor**	**1939.622**	**0.001**	**573**	**3.385**	**0.992**	**0.991**	**0.056**	**0.055**
2nd order 6F	6472.337	0.001	588	11.01	0.965	0.962	0.114	0.111
2nd order 7F	2574.417	0.001	587	4.386	0.988	0.987	0.066	0.065

Table 9. Standardized Coefficients CFA Unidimensional Model

		Working		Non-Working		Imputed	
Observed	Latent	β	*SE*	β	*SE*	β	*SE*
D1.1	General	0.61	0.03	0.63	0.03	0.61	0.02
D1.2	General	0.58	0.03	0.61	0.03	0.59	0.02
D1.3	General	0.59	0.03	0.56	0.04	0.58	0.02
D1.4	General	0.59	0.03	0.57	0.04	0.58	0.02
D1.5	General	0.61	0.03	0.55	0.04	0.59	0.02
D1.6	General	0.55	0.03	0.57	0.04	0.55	0.02
D2.1	General	0.56	0.03	0.62	0.04	0.60	0.02
D2.2	General	0.58	0.03	0.70	0.03	0.64	0.02
D2.3	General	0.68	0.03	0.71	0.03	0.70	0.02
D2.4	General	0.64	0.03	0.64	0.03	0.65	0.02
D2.5	General	0.63	0.03	0.69	0.03	0.65	0.02
D3.1	General	0.72	0.03	0.83	0.02	0.78	0.02
D3.2	General	0.71	0.03	0.82	0.02	0.77	0.02
D3.3	General	0.47	0.03	0.55	0.04	0.50	0.03
D3.4	General	0.42	0.04	0.52	0.04	0.45	0.03
D4.1	General	0.63	0.03	0.65	0.03	0.64	0.02
D4.2	General	0.59	0.03	0.60	0.04	0.59	0.02
D4.3	General	0.60	0.03	0.63	0.03	0.60	0.02
D4.4	General	0.60	0.03	0.61	0.04	0.60	0.02
D4.5	General	0.43	0.04	0.45	0.05	0.44	0.03
D5.1	General	0.85	0.01	0.91	0.01	0.88	0.01
D5.2	General	0.81	0.01	0.89	0.01	0.85	0.01
D5.3	General	0.83	0.01	0.89	0.01	0.86	0.01
D5.4	General	0.79	0.02	0.80	0.02	0.80	0.01
D5.5	General	0.79	0.02	N/A	N/A	0.74	0.01
D5.6	General	0.86	0.01	N/A	N/A	0.83	0.01
D5.7	General	0.93	0.01	N/A	N/A	0.86	0.01
D5.8	General	0.88	0.01	N/A	N/A	0.72	0.01
D6.1	General	0.67	0.02	0.64	0.03	0.66	0.02
D6.2	General	0.67	0.02	0.64	0.03	0.66	0.02
D6.3	General	0.52	0.03	0.57	0.04	0.54	0.02
D6.4	General	0.41	0.03	0.41	0.05	0.41	0.03
D6.5	General	0.56	0.03	0.58	0.04	0.57	0.02
D6.6	General	0.40	0.04	0.41	0.05	0.42	0.03
D6.7	General	0.47	0.03	0.47	0.04	0.47	0.03
D6.8	General	0.57	0.03	0.59	0.04	0.57	0.02

Note. CFA = Confirmatory Factor Analysis

Table 10. Standardized Coefficients CFA 6 Factor Correlated Traits Model

		Working		Non-Working		Imputed	
Observed	Latent	β	*SE*	β	*SE*	β	*SE*
D1.1	Cognition	0.76	0.02	0.79	0.03	0.77	0.02
D1.2	Cognition	0.72	0.03	0.76	0.03	0.74	0.02
D1.3	Cognition	0.74	0.03	0.70	0.04	0.73	0.02
D1.4	Cognition	0.73	0.03	0.72	0.04	0.74	0.02
D1.5	Cognition	0.74	0.03	0.69	0.04	0.74	0.02
D1.6	Cognition	0.69	0.03	0.72	0.04	0.70	0.02
D2.1	Mobility	0.76	0.03	0.76	0.03	0.77	0.02
D2.2	Mobility	0.76	0.03	0.84	0.03	0.81	0.02
D2.3	Mobility	0.87	0.03	0.86	0.03	0.88	0.02
D2.4	Mobility	0.87	0.03	0.82	0.03	0.85	0.02
D2.5	Mobility	0.81	0.03	0.81	0.03	0.81	0.02
D3.1	Self-Care	0.90	0.03	0.94	0.02	0.93	0.02
D3.2	Self-Care	0.92	0.03	0.92	0.02	0.92	0.02
D3.3	Self-Care	0.68	0.04	0.72	0.04	0.69	0.03
D3.4	Self-Care	0.60	0.04	0.68	0.05	0.62	0.03
D4.1	Getting Along	0.83	0.02	0.81	0.03	0.82	0.02
D4.2	Getting Along	0.78	0.02	0.75	0.03	0.77	0.02
D4.3	Getting Along	0.78	0.03	0.78	0.03	0.77	0.02
D4.4	Getting Along	0.79	0.02	0.77	0.03	0.78	0.02
D4.5	Getting Along	0.56	0.04	0.56	0.05	0.57	0.03
D5.1	Life Activities	0.90	0.01	0.95	0.01	0.92	0.01
D5.2	Life Activities	0.87	0.01	0.94	0.01	0.91	0.01
D5.3	Life Activities	0.88	0.01	0.93	0.01	0.90	0.01
D5.4	Life Activities	0.85	0.01	0.87	0.02	0.86	0.01
D5.5	Life Activities	0.84	0.01	N/A	N/A	0.81	0.01
D5.6	Life Activities	0.90	0.01	N/A	N/A	0.88	0.01
D5.7	Life Activities	0.95	0.01	N/A	N/A	0.91	0.01
D5.8	Life Activities	0.91	0.01	N/A	N/A	0.79	0.01
D6.1	Participation	0.82	0.02	0.78	0.03	0.81	0.02
D6.2	Participation	0.81	0.02	0.78	0.03	0.81	0.02
D6.3	Participation	0.64	0.03	0.70	0.04	0.68	0.02
D6.4	Participation	0.51	0.04	0.51	0.05	0.52	0.03
D6.5	Participation	0.69	0.03	0.72	0.04	0.71	0.02
D6.6	Participation	0.49	0.05	0.53	0.05	0.53	0.03
D6.7	Participation	0.59	0.04	0.60	0.04	0.59	0.03
D6.8	Participation	0.69	0.03	0.74	0.04	0.71	0.02

Note. CFA = Confirmatory Factor Analysis

Table 11. Standardized Coefficients CFA 7 Factor Correlated Traits Model

		Working		Non-Working		Imputed	
Observed	Latent	β	*SE*	β	*SE*	β	*SE*
D1.1	Cognition	0.76	0.02	N/A	N/A	0.76	0.02
D1.2	Cognition	0.72	0.03	N/A	N/A	0.74	0.02
D1.3	Cognition	0.74	0.03	N/A	N/A	0.73	0.02
D1.4	Cognition	0.73	0.03	N/A	N/A	0.74	0.02
D1.5	Cognition	0.74	0.03	N/A	N/A	0.73	0.02
D1.6	Cognition	0.69	0.03	N/A	N/A	0.70	0.02
D2.1	Mobility	0.76	0.03	N/A	N/A	0.77	0.02
D2.2	Mobility	0.76	0.03	N/A	N/A	0.81	0.02
D2.3	Mobility	0.87	0.03	N/A	N/A	0.87	0.02
D2.4	Mobility	0.86	0.03	N/A	N/A	0.85	0.02
D2.5	Mobility	0.81	0.03	N/A	N/A	0.81	0.02
D3.1	Self-Care	0.90	0.03	N/A	N/A	0.93	0.02
D3.2	Self-Care	0.92	0.03	N/A	N/A	0.92	0.02
D3.3	Self-Care	0.68	0.04	N/A	N/A	0.70	0.03
D3.4	Self-Care	0.60	0.04	N/A	N/A	0.63	0.03
D4.1	Getting Along	0.83	0.02	N/A	N/A	0.82	0.02
D4.2	Getting Along	0.78	0.02	N/A	N/A	0.77	0.02
D4.3	Getting Along	0.78	0.03	N/A	N/A	0.77	0.02
D4.4	Getting Along	0.79	0.02	N/A	N/A	0.78	0.02
D4.5	Getting Along	0.56	0.04	N/A	N/A	0.57	0.03
D5.1	LA Household	0.92	0.01	N/A	N/A	0.94	0.01
D5.2	LA Household	0.91	0.01	N/A	N/A	0.92	0.01
D5.3	LA Household	0.90	0.01	N/A	N/A	0.92	0.01
D5.4	LA Household	0.90	0.01	N/A	N/A	0.89	0.01
D5.5	LA Working	0.89	0.01	N/A	N/A	0.87	0.01
D5.6	LA Working	0.93	0.01	N/A	N/A	0.92	0.01
D5.7	LA Working	0.96	0.01	N/A	N/A	0.94	0.01
D5.8	LA Working	0.93	0.01	N/A	N/A	0.86	0.01
D6.1	Participation	0.82	0.02	N/A	N/A	0.81	0.02
D6.2	Participation	0.81	0.02	N/A	N/A	0.81	0.02
D6.3	Participation	0.64	0.03	N/A	N/A	0.68	0.02
D6.4	Participation	0.51	0.04	N/A	N/A	0.52	0.03
D6.5	Participation	0.69	0.03	N/A	N/A	0.71	0.02
D6.6	Participation	0.49	0.05	N/A	N/A	0.53	0.03
D6.7	Participation	0.59	0.04	N/A	N/A	0.59	0.03
D6.8	Participation	0.69	0.03	N/A	N/A	0.71	0.02

Note. CFA = Confirmatory Factor Analysis

Table 12. Standardized Coefficients 2nd order 6 Factor Model

		Working		Non-Working		Imputed	
Observed	Latent	β	*SE*	β	*SE*	β	*SE*
D1.1	Cognition	0.76	0.02	0.79	0.03	0.77	0.02
D1.2	Cognition	0.72	0.03	0.76	0.03	0.74	0.02
D1.3	Cognition	0.74	0.03	0.70	0.04	0.73	0.02
D1.4	Cognition	0.73	0.03	0.72	0.04	0.74	0.02
D1.5	Cognition	0.75	0.03	0.69	0.04	0.74	0.02
D1.6	Cognition	0.68	0.03	0.72	0.04	0.70	0.02
D2.1	Mobility	0.75	0.03	0.76	0.03	0.77	0.02
D2.2	Mobility	0.75	0.03	0.84	0.03	0.81	0.02
D2.3	Mobility	0.87	0.03	0.86	0.03	0.88	0.02
D2.4	Mobility	0.87	0.03	0.81	0.03	0.85	0.02
D2.5	Mobility	0.81	0.03	0.82	0.03	0.81	0.02
D3.1	Self-Care	0.90	0.03	0.94	0.02	0.93	0.02
D3.2	Self-Care	0.92	0.03	0.92	0.02	0.92	0.02
D3.3	Self-Care	0.68	0.04	0.72	0.04	0.70	0.03
D3.4	Self-Care	0.60	0.04	0.68	0.05	0.62	0.03
D4.1	Getting Along	0.83	0.02	0.81	0.03	0.83	0.02
D4.2	Getting Along	0.78	0.02	0.75	0.03	0.77	0.02
D4.3	Getting Along	0.78	0.03	0.79	0.03	0.77	0.02
D4.4	Getting Along	0.78	0.02	0.76	0.03	0.77	0.02
D4.5	Getting Along	0.56	0.04	0.56	0.05	0.57	0.03
D5.1	Life Activities	0.90	0.01	0.95	0.01	0.92	0.01
D5.2	Life Activities	0.87	0.01	0.94	0.01	0.91	0.01
D5.3	Life Activities	0.88	0.01	0.93	0.01	0.90	0.01
D5.4	Life Activities	0.85	0.01	0.87	0.02	0.86	0.01
D5.5	Life Activities	0.84	0.01	N/A	N/A	0.81	0.01
D5.6	Life Activities	0.90	0.01	N/A	N/A	0.88	0.01
D5.7	Life Activities	0.95	0.01	N/A	N/A	0.91	0.01
D5.8	Life Activities	0.91	0.01	N/A	N/A	0.79	0.01
D6.1	Participation	0.82	0.02	0.78	0.03	0.81	0.02
D6.2	Participation	0.81	0.02	0.78	0.03	0.81	0.02
D6.3	Participation	0.64	0.03	0.70	0.04	0.68	0.02
D6.4	Participation	0.50	0.04	0.51	0.05	0.52	0.03
D6.5	Participation	0.69	0.03	0.72	0.04	0.71	0.02
D6.6	Participation	0.49	0.05	0.53	0.05	0.53	0.03
D6.7	Participation	0.59	0.04	0.60	0.04	0.59	0.03
D6.8	Participation	0.69	0.03	0.74	0.04	0.71	0.02
SOF	Cognition	0.84	0.02	0.76	0.04	0.81	0.02
SOF	Mobility	0.72	0.03	0.77	0.03	0.75	0.02
SOF	Self-Care	0.72	0.03	0.74	0.03	0.73	0.02
SOF	Getting Along	0.77	0.03	0.80	0.03	0.78	0.02
SOF	Life Activities	0.67	0.02	0.73	0.03	0.63	0.02
SOF	Participation	0.85	0.02	0.77	0.04	0.82	0.02

Note. CFA = Confirmatory Factor Analysis; SOF = Second Order Factor

Table 13. Standardized Coefficients 2nd order 7 Factor Model

		Working		Non-Working		Imputed	
Observed	Latent	β	*SE*	β	*SE*	β	*SE*
D1.1	Cognition	0.76	0.02	N/A	N/A	0.76	0.02
D1.2	Cognition	0.72	0.03	N/A	N/A	0.74	0.02
D1.3	Cognition	0.74	0.03	N/A	N/A	0.73	0.02
D1.4	Cognition	0.73	0.03	N/A	N/A	0.74	0.02
D1.5	Cognition	0.75	0.03	N/A	N/A	0.74	0.02
D1.6	Cognition	0.68	0.03	N/A	N/A	0.70	0.02
D2.1	Mobility	0.76	0.03	N/A	N/A	0.77	0.02
D2.2	Mobility	0.76	0.03	N/A	N/A	0.81	0.02
D2.3	Mobility	0.87	0.03	N/A	N/A	0.88	0.02
D2.4	Mobility	0.87	0.03	N/A	N/A	0.85	0.02
D2.5	Mobility	0.81	0.03	N/A	N/A	0.81	0.02
D3.1	Self-Care	0.90	0.03	N/A	N/A	0.93	0.02
D3.2	Self-Care	0.92	0.03	N/A	N/A	0.92	0.02
D3.3	Self-Care	0.68	0.04	N/A	N/A	0.70	0.03
D3.4	Self-Care	0.60	0.04	N/A	N/A	0.63	0.03
D4.1	Getting Along	0.83	0.02	N/A	N/A	0.83	0.02
D4.2	Getting Along	0.78	0.02	N/A	N/A	0.77	0.02
D4.3	Getting Along	0.78	0.03	N/A	N/A	0.78	0.02
D4.4	Getting Along	0.78	0.02	N/A	N/A	0.77	0.02
D4.5	Getting Along	0.56	0.04	N/A	N/A	0.57	0.03
D5.1	LA Household	0.92	0.01	N/A	N/A	0.94	0.01
D5.2	LA Household	0.91	0.01	N/A	N/A	0.92	0.01
D5.3	LA Household	0.90	0.01	N/A	N/A	0.92	0.01
D5.4	LA Household	0.89	0.01	N/A	N/A	0.89	0.01
D5.5	LA Working	0.89	0.01	N/A	N/A	0.87	0.01
D5.6	LA Working	0.93	0.01	N/A	N/A	0.92	0.01
D5.7	LA Working	0.96	0.01	N/A	N/A	0.94	0.01
D5.8	LA Working	0.93	0.01	N/A	N/A	0.86	0.01
D6.1	Participation	0.83	0.02	N/A	N/A	0.81	0.02
D6.2	Participation	0.81	0.02	N/A	N/A	0.81	0.02
D6.3	Participation	0.64	0.03	N/A	N/A	0.68	0.02
D6.4	Participation	0.50	0.04	N/A	N/A	0.51	0.03
D6.5	Participation	0.69	0.03	N/A	N/A	0.71	0.02
D6.6	Participation	0.49	0.05	N/A	N/A	0.53	0.03
D6.7	Participation	0.58	0.04	N/A	N/A	0.59	0.03
D6.8	Participation	0.69	0.03	N/A	N/A	0.71	0.03
SOF	Cognition	0.84	0.02	N/A	N/A	0.81	0.02
SOF	Mobility	0.72	0.03	N/A	N/A	0.75	0.02
SOF	Self-Care	0.72	0.03	N/A	N/A	0.73	0.02
SOF	Getting Along	0.77	0.03	N/A	N/A	0.79	0.02
SOF	LA Household	0.69	0.03	N/A	N/A	0.72	0.02
SOF	LA Working	0.59	0.03	N/A	N/A	0.45	0.03
SOF	Participation	0.85	0.02	N/A	N/A	0.82	0.02

Note. CFA = Confirmatory Factor Analysis; SOF = Second Order Factor
