# Supplementary material for: Support for the higher-order factor structure of the WHODAS 2.0 self-report version in a Dutch outpatient psychiatric setting
Source: Qual Life Res. 2021 Jun 12;30(10):2939–49. doi: 10.1007/s11136-021-02880-8 (PMC8481147; doi:10.1007/s11136-021-02880-8)
Supplement: Supplementary file 2 — Supplementary file2 (PDF 92 KB) [file 11136_2021_2880_MOESM2_ESM.pdf]

Online supplement B

Item descriptive statistics and Likert scale distributions

Title: Support WHODAS 2.0 factor structure

Journal: Quality of Life Research

Authors: Guido L. Williams, Edwin de Beurs, Philip Spinhoven, Gerard Flens, Muirne C. S. Paap
Corresponding author: Dimence Group, Dimence foundation, Specialized Assessment and Treatment Division, Department of Digital Mental Healthcare, Deventer, The Netherlands; g.williams@thubble.nl; ORCID 0000-0001-8008-5750

Table 14. Item Descriptive Statistics WHODAS 2.0 (n = 770)

Item	*Mean*	*sd*	*median*	*mad*[a]	*skew*	*kurtosis*	*se*
D1.1	2.8	1.3	3	1.5	0.1	-1.1	0.05
D1.2	2.9	1.3	3	1.5	0.1	-1.2	0.05
D1.3	2.9	1.2	3	1.5	-0.0	-1.0	0.04
D1.4	2.4	1.4	2	1.5	0.5	-1.0	0.05
D1.5	2.1	1.1	2	1.5	0.8	-0.3	0.04
D1.6	2.5	1.3	2	1.5	0.4	-1.0	0.05
D2.1	2.3	1.4	2	1.5	0.7	-0.9	0.05
D2.2	1.9	1.2	1	0.0	1.1	-0.0	0.04
D2.3	1.7	1.1	1	0.0	1.4	0.9	0.04
D2.4	2.3	1.3	2	1.5	0.6	-0.9	0.05
D2.5	2.0	1.4	1	0.0	1.1	-0.1	0.05
D3.1	1.6	1.0	1	0.0	1.9	2.7	0.04
D3.2	1.5	1.0	1	0.0	1.9	2.8	0.04
D3.3	2.0	1.2	2	1.5	1.0	-0.1	0.04
D3.4	2.3	1.4	2	1.5	0.7	-0.9	0.05
D4.1	2.4	1.3	2	1.5	0.6	-0.8	0.05
D4.2	2.6	1.3	2	1.5	0.3	-1.0	0.05
D4.3	2.1	1.1	2	1.5	0.7	-0.4	0.04
D4.4	2.9	1.5	3	3.0	0.1	-1.5	0.06
D4.5	2.6	1.6	2	1.5	0.4	-1.4	0.06
D5.1	2.7	1.3	3	1.5	0.3	-1.1	0.05
D5.2	2.4	1.3	2	1.5	0.5	-0.9	0.05
D5.3	2.7	1.3	3	1.5	0.2	-1.1	0.05
D5.4	2.7	1.4	3	1.5	0.2	-1.2	0.05
D5.5[b]	3.2	1.4	3	1.5	-0.1	-1.3	0.06
D5.6[b]	3.0	1.4	3	1.5	0.1	-1.3	0.06
D5.7[b]	3.0	1.5	3	1.5	0.0	-1.4	0.06
D5.8[b]	3.0	1.5	3	1.5	0.1	-1.4	0.06
D6.1	3.0	1.4	3	1.5	-0.8	-1.2	0.05
D6.2	2.8	1.4	3	1.5	0.1	-1.3	0.05
D6.3	2.4	1.3	2	1.5	0.4	-1.1	0.05
D6.4	3.0	1.2	3	1.5	0.0	-1.1	0.05
D6.5	3.3	1.3	4	1.5	-0.3	-0.9	0.05

D6.6	2.0	1.3	1	0.0	1.0	-0.4	0.05
D6.7	2.7	1.3	3	1.5	0.2	-1.1	0.05
D6.8	3.3	1.3	3	1.5	-0.3	-1.1	0.05

[a] median absolute deviation
[b] n = 483

Table 15. Percentages Table Likert Scale Distributions WHODAS 2.0 (n = 770)

	% Scoring Categories Endorsed				
	1	2	3	4	5
Item	None	Mild	Moderate	Severe	Extreme
D1.1	17.5	26.9	21.8	22.7	11.0
D1.2	19.2	24.7	20.1	22.2	13.8
D1.3	14.8	23.4	26.6	25.2	10.0
D1.4	35.1	22.2	18.7	14.0	10.0
D1.5	34.0	35.2	16.6	11.7	2.6
D1.6	29.0	27.1	18.1	17.3	8.8
D2.1	46.8	17.1	11.6	13.5	11.0
D2.2	55.6	19.0	10.5	12.0	3.0
D2.3	62.2	17.1	11.0	7.5	2.1
D2.4	36.8	25.6	14.9	16.6	6.1
D2.5	56.6	16.5	8.6	9.0	9.4
D3.1	70.3	14.8	6.6	5.7	2.6
D3.2	72.1	13.4	6.8	5.8	2.0
D3.3	47.5	24.2	13.6	10.5	4.0
D3.4	42.3	18.8	15.3	12.9	10.7
D4.1	31.8	30.1	15.3	14.2	8.6
D4.2	26.4	26.1	21.3	18.3	7.9
D4.3	37.7	29.7	18.6	11.3	2.7
D4.4	28.8	16.6	16.5	15.5	22.6
D4.5	38.1	16.1	14.2	12.3	19.4
D5.1	22.1	27.5	21.0	19.7	9.6
D5.2	30.8	28.2	17.0	16.5	7.5
D5.3	23.1	24.3	21.3	20.1	11.2
D5.4	24.6	24.3	18.6	20.1	12.5
D5.5[a]	13.9	23.8	17.6	22.8	22.2
D5.6[a]	17.6	25.3	17.0	18.5	21.6
D5.7[a]	20.9	20.3	19.5	17.0	22.4
D5.8[a]	21.3	21.3	17.4	17.0	23.0
D6.1	19.2	19.2	20.4	25.8	15.3
D6.2	25.6	19.6	20.5	23.3	11.0
D6.3	33.6	23.3	17.9	19.2	6.0
D6.4	14.6	24.6	23.4	25.6	12.0

D6.5	9.9	17.0	23.0	28.6	22.0
D6.6	51.7	18.6	11.3	11.0	7.4
D6.7	23.6	24.4	22.0	20.1	9.9
D6.8	13.4	16	22.1	25.1	23.5

[a] n = 483
